# Antibiotic Susceptibility Patterns of Salmonella Typhi and Salmonella Paratyphi in a Tertiary Care Hospital in Islamabad

**DOI:** 10.7759/cureus.10228

**Published:** 2020-09-03

**Authors:** Masab Umair, Shajee Ahmad Siddiqui

**Affiliations:** 1 General (Internal) Medicine, Pakistan Institute of Medical Sciences, Shaheed Zulfiqar Ali Bhutto Medical University, Islamabad, PAK

**Keywords:** salmonella, typhoid, paratyphoid, enteric fever, multi drug resistant, mdr, extensively drug resistant, xdr

## Abstract

Background

Enteric fever is a serious public health problem in Pakistan. Growing problem of drug-resistant Salmonella strains and outbreak of ceftriaxone-resistant Salmonella typhi in Hyderabad during 2016-2017 is concerning. This study aimed to determine the antibiogram profile of Salmonella typhi and Salmonella paratyphi isolated from blood cultures of patients presenting in Pakistan Institute of Medical Sciences (PIMS), Islamabad.

Materials and methods

A retrospective cross-sectional study conducted in PIMS. A case of enteric fever was defined as a patient with blood culture positive for either S. typhi or S. paratyphi. Demographics and antibiogram profile of the 664 cases who presented during 2012-2018 were included in this study.

Results

Out of 664 cases, S. typhi was isolated from 528 and S. paratyphi was isolated from 136 cases. Males accounted for the majority of the cases (n = 440, 66.3%). Clustering of the cases was observed in young adults (18-25 years). Incidence was highest during months of summer and monsoon (May-September). Most of the S. typhi isolates were resistant to the first-line antibiotics (amoxicillin 57.6%, co-trimoxazole 61.4%, chloramphenicol 46.9%) and ciprofloxacin (62.7%). Antibiotic resistance rates were lowest for imipenem (3.8%) and ceftriaxone (4.4%). Among S. typhi isolates tested for all first-line antibiotics, 44.6% (149/334) were multidrug-resistant (MDR). In contrast, only 12.2% (11/90) of the S. paratyphi isolates were MDR. 0.7% (2/283) of the tested S. typhi isolates were extensively drug-resistant (XDR). XDR strains were sensitive to imipenem. There was an overall reduction in first-line antibiotic resistance rates from 2012 to 2018.

Conclusion

S. typhi accounted for the majority of the cases of enteric fever. Most S. typhi isolates were resistant to first-line antibiotics. S. paratyphi exhibited lower antibiotic resistance rates. This study recommends third-generation cephalosporins for empirical therapy and for treatment of MDR cases of enteric fever. Imipenem should be reserved for the treatment of XDR Salmonella cases. A decreasing trend in first-line antibiotic resistance rates over time is promising. Antibiotic stewardship is the need of the hour.

## Introduction

Enteric fever is a disease of intestinal tract caused by Salmonella typhi (typhoid fever) and Salmonella paratyphi (paratyphoid fever). Patients present with fever, malaise, abdominal pain, and constipation. Annually, 21.6 million cases of typhoid fever with 250,000 deaths and 5.4 million cases of paratyphoid fever occur worldwide [1]. Approximately 80% of the cases and deaths occur in Asia [1]. Incidence of enteric fever in India is from 102-2219/100,000 population [1]. Enteric fever is a major health problem in Pakistan. Estimated annual incidence of S. typhi and S. paratyphi among children in Karachi is 451/100,000 and 76/100,000, respectively [2]. Untreated typhoid fever carries high morbidity and mortality. Without appropriate treatment, typhoid fever carries estimated mortality rate of 30% [1].

Intestinal perforation is a dreaded complication of typhoid fever [3]. Other complications of typhoid fever include but are not limited to pneumonia, meningitis, endocarditis, osteomyelitis, and arthritis [3]. Presenting symptoms of typhoid fever, therefore, may relate to various organs often resulting in misdiagnosis [3]. Isolation of the organism from the blood culture is vital for the diagnosis of typhoid fever [3].

Ampicillin, chloramphenicol, and co-trimoxazole used to be the first-line antibiotics for the treatment of enteric fever [4]. Since the late 1980s, multidrug-resistant strains of Salmonella (MDR, resistant to all first-line antibiotics) have been reported [5]. For quinolone sensitive strains of MDR typhoid, fluoroquinolone is the drug of choice (WHO guidelines 2003) [1]. However, the widespread use of fluoroquinolones has led to the emergence of nalidixic acid-resistant strains with reduced sensitivity to fluoroquinolones in Nepal [5]. In Kolkata and Karachi, approximately 60% of typhoid fever isolates were found to be nalidixic acid-resistant [6]. This has led to increased use of third-generation cephalosporins. However, third-generation cephalosporin-resistant Salmonella strains have been reported in Nepal and India [1,5]. There has been an outbreak of extensively drug-resistant (XDR) S. typhi in Pakistan during 2016-2017 [7]. This situation is very alarming; clinicians are running out of treatment options and treatment costs have escalated.

Since the emergence of drug-resistant strains of Salmonella, first-line antibiotics fell out of favor and were not frequently used for this illness lately. Interestingly, there have been recent reports of first-line antibiotic susceptible Salmonella strains in Nepal and India [5,8]. This re-emergence of first-line antibiotic sensitive Salmonella strains is promising and highlights the importance of continuous antibiogram surveillance.

The aim of this study is to determine the antibiogram profile of S. typhi and S. paratyphi isolated from blood cultures of patients presenting in Pakistan Institute of Medical Sciences. The rationale is to propose appropriate empirical antibiotics for enteric fever in view of increasing drug resistance and evolving antibiograms.

## Materials and methods

This is a retrospective cross-sectional study that was conducted in Pakistan Institute of Medical Sciences. A case of enteric fever was defined as a patient with blood culture positive for either S. typhi or S. paratyphi. Non-probability consecutive sampling technique was used. This study included the patients aged 12 years and above with blood cultures positive for either S. typhi or S. paratyphi during 2012-2018. Patient’s age, gender, date of admission, Salmonella serotype isolated and antibiotic sensitivity profile tested for amoxicillin, azithromycin, cefixime, ceftriaxone, chloramphenicol, ciprofloxacin, cotrimoxazole, imipenem and nalidixic acid were recorded and analysed. MDR was defined as resistance to amoxicillin, chloramphenicol and co-trimoxazole (i.e., first-line antibiotics). XDR was defined as resistance to ciprofloxacin and ceftriaxone (i.e., fluoroquinolones and third-generation cephalosporins) in addition to first-line antibiotic resistance (amoxicillin, chloramphenicol and co-trimoxazole).

## Results

Out of 664 cases included in the study, S. typhi was isolated from 528 (79.5%) and S. paratyphi was isolated from 136 (20.5%) cases. 440 (66.3%) were males and 224 (33.7%) were females with male to female ratio ≈ 2:1. Mean age was 25.5 years (range 12-81 years). Most of the cases belonged to 18-25 years age group. Age and gender distribution of the cases of S. typhi and S. paratyphi are shown in Table 1. 

 

**Table 1:** Age and gender distribution of cases of Salmonella typhi and Salmonella paratyphi

	Salmonella typhi	Salmonella paratyphi	
	n (%)	n (%)	Total
Distribution by age			
Adolescents (12-17 years)	146 (27.7)	26 (19.1)	172
Young adults (18-35 years)	306 (58)	89 (65.4)	395
Middle aged adults (36-55 years)	52 (9.8)	16 (11.8)	68
Older adults (55 years and above)	24 (4.5)	5 (3.7)	29
Total	528	136	
Distribution by gender			
Male	342 (64.8)	98 (72.1)	440
Female	186 (35.2)	38 (27.9)	224
Total	528	136	

**Figure 1:**
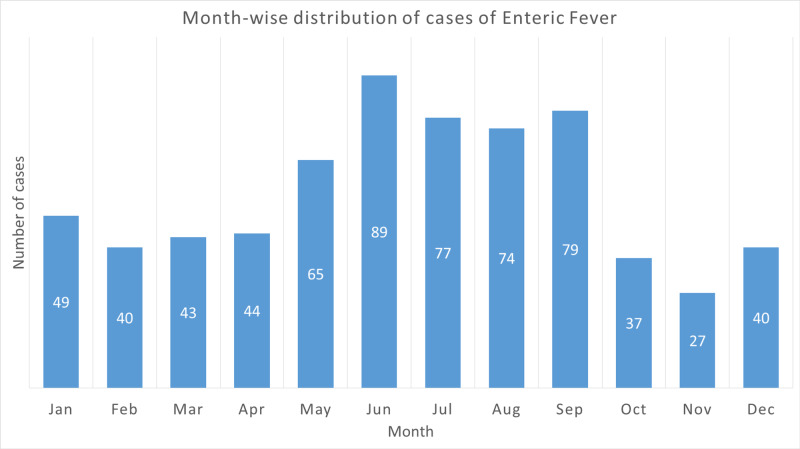
Month-wise distribution of cases of enteric fever

Clustering of the cases was observed during months of summer and monsoon, that is, May-September (Figure 1).

Most of the S. typhi isolates were resistant to first-line antibiotics and ciprofloxacin. In general, antibiotic resistance rates were lower in S. paratyphi with exception of azithromycin and ciprofloxacin. Proportion of MDR and XDR strains was higher in S. typhi (Table 2). 

**Table 2:** Antibiotic resistance patterns of Salmonella typhi and Salmonella paratyphi isolates ^a^Tested simultaneously for amoxicillin, chloramphenicol and co-trimoxazole. ^b^Tested simultaneously for amoxicillin, chloramphenicol, co-trimoxazole, ciprofloxacin and ceftriaxone. MDR, multidrug-resistant; XDR, extensively drug-resistant.

	Salmonella typhi		Salmonella paratyphi	
	Cases tested	Resistant	Cases tested	Resistant
	n	n (%)	n	n (%)
Amoxicillin	429	247 (57.6)	111	32 (28.8)
Azithromycin	322	204 (63.4)	88	65 (73.9)
Cefixime	240	17 (7.1)	42	1 (2.4)
Ceftriaxone	481	21 (4.4 )	123	2 (1.6)
Chloramphenicol	471	221 (46.9)	125	25 (20)
Ciprofloxacin	475	298 (62.7)	122	86 (70.5)
Co-trimoxazole	440	270 (61.4)	117	42 (35.9)
Imipenem	373	14 (3.8)	103	3 (2.9)
Nalidixic acid	442	408 (92.3)	124	114 (91.9)
MDR tested ^a^	334	149 (44.6)	90	11 (12.2)
XDR tested ^b^	283	2 (0.7)	75	0 (0)

Most of the MDR Salmonella strains were sensitive to third-generation cephalosporins and imipenem. XDR S. typhi strains were all imipenem sensitive (Table 3). 

 

**Table 3:** Antibiotic susceptibility pattern of MDR and XDR strains of Salmonella isolates MDR, multidrug-resistant; XDR, extensively drug-resistant

	Salmonella typhi		Salmonella paratyphi	
	Cases tested	Sensitive	Cases tested	Sensitive
	n	n (%)	n	n (%)
	Multidrug-resistant			
Azithromycin	100	29 (29)	6	1 (16.7)
Cefixime	55	51 (92.7)	6	6 (100)
Ceftriaxone	142	138 (97.2)	10	10 (100)
Ciprofloxacin	142	10 (7)	11	0 (0)
Imipenem	97	91 (93.8)	10	9 (90)
	Extensively drug-resistant			
Azithromycin	1	1 (100)	-	-
Imipenem	2	2 (100)	-	-

**Figure 2:**
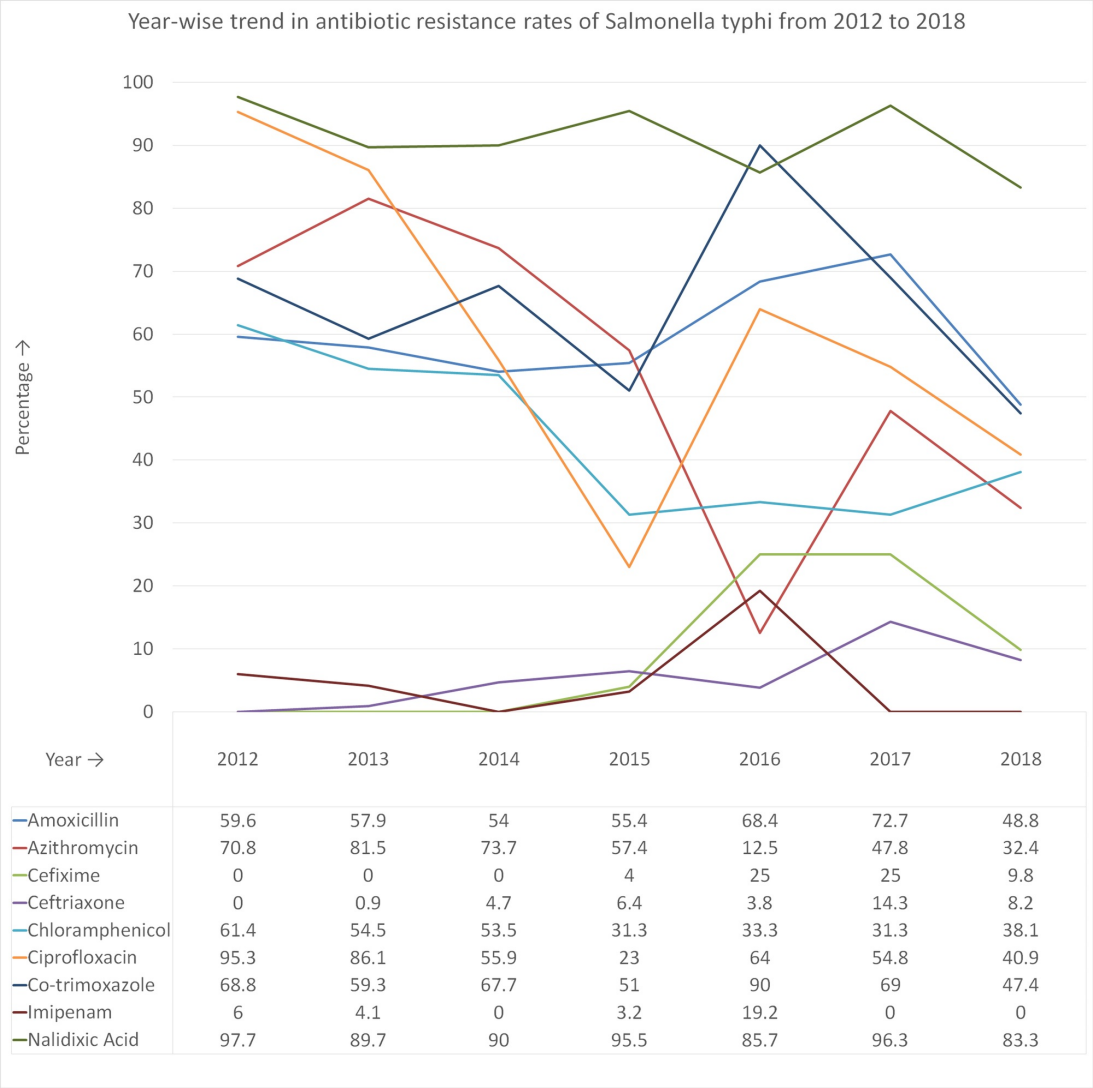
Year-wise trend in antibiotic resistance rates of Salmonella typhi from 2012 to 2018

There has been an overall decrease in first-line antibiotic resistance rates and an increase in third-generation cephalosporins resistance rates from 2012 to 2018 in S. typhi isolates. There was a sharp rise in resistance rates of most antibiotics during 2016 and 2017 (Figure 2). 

## Discussion

Enteric fever is a foodborne and waterborne infectious disease caused by S. typhi (typhoid fever) and S. paratyphi (paratyphoid fever) [9]. Untreated infections can result in serious morbidity and even mortality. Most of the cases of enteric fever occur in Asia [1]. Enteric fever is endemic in the Indian sub-continent [10]. It is a major health problem in Pakistan. Antibiotic resistance is a major problem in the management of enteric fever. MDR and XDR cases of enteric fever have been reported [5,11,12]. The largest outbreak of ceftriaxone-resistant S. typhi was reported in Pakistan during 2017-2018 [12]. The knowledge of the local Salmonella antibiogram and antibiotic stewardship is crucial for the management of enteric fever. This study aimed to determine the antibiogram profile and compared year-wise trends in antibiotic resistance patterns of the cases of S. typhi and S. paratyphi in Pakistan Institute of Medical Sciences (PIMS) from 2012 to 2018.

In the present study, S. typhi was the predominant isolate from the patients with enteric fever in agreement with the previous studies [2,13,14,15]. Most of the cases were males. Other studies also reported a similar gender distribution [1,13,16,17]. Plausible explanation for this male predominance is the fact that males have more outdoor exposure and are more likely to consume street foods as compared to females in Pakistani society [1,13,17]. Street food consumption is an important risk factor for enteric fever [18]. A study conducted in Karachi reported a high prevalence of Salmonella carriage among food handlers working in food streets [19]. Analysis of age distribution reveals the clustering of cases in young adults (18-35 years). Sharvani et al. reported similar age distribution of the cases [1]. Young adults are more likely to be exposed to the pathogen because of the similar reasons that explain male prevalence. Clustering of cases was also observed during months of summer and monsoon in the present study. Khan et al. also reported a higher incidence during the summer season and attributed this trend to increased consumption of commercially prepared ice, chilled drinks and floods [10]. Sur et al. and Mohanty et al. reported a similar seasonal trend in India [16,20].

Majority of the S. typhi isolates were resistant to all first-line antibiotics (amoxicillin, co-trimoxazole and chloramphenicol). Most of the S. typhi isolates including MDR strains were sensitive to cephalosporins and imipenem but resistant to ciprofloxacin and azithromycin. Qamar et al. and Khan et al. also reported a high prevalence of MDR Salmonella strains and a similar antibiotic resistance profile in Karachi and Hyderabad [2,13]. In contrast to our findings, Laghari et al. reported lower ceftriaxone and cefixime sensitivity rates, higher first-line antibiotic sensitivity rates and very low proportion of MDR strains among pediatric cases of S. typhi in Jamshoro [21]. Studies conducted in India and Nepal reported lower first-line antibiotic resistance rates and lower proportion of MDR strains among S. typhi [1,22].

In this study, two XDR cases of S. typhi were identified; both were sensitive to imipenem, a carbapenem antibiotic. One of these cases was tested for and found to be sensitive to azithromycin as well. A study conducted on XDR S. typhi in Northern Pakistan reported antibiotic sensitivity only to azithromycin and meropenem [11]. Yousafzai et al. investigated the outbreak of ceftriaxone-resistant S. typhi in Hyderabad during 2016-2017 and reported a similar antibiotic sensitivity profile, that is, azithromycin, imipenem and meropenem [12].

In general, first-line antibiotic resistance rates were much lower among cases of S. paratyphi as compared to S. typhi thus accounting for lower proportion of MDR S. paratyphi in this study. This trend is consistent with the findings of Qamar et al. and Laghari et al. [13,21]. Furthermore, most of the S. paratyphi isolates were resistant to azithromycin, nalidixic acid, and ciprofloxacin but sensitive to cephalosporins and imipenem in our study. Sharvani et al. reported a similar trend in cephalosporin, ciprofloxacin and nalidixic acid resistance among cases of S. paratyphi in India [1]. No XDR strain of S. paratyphi was identified in our study. 

Analysis of year-wise trends in antibiotic resistance profile of S. typhi from 2012 to 2018 reveals a sharp rise in resistance rates of most antibiotics during 2016 and 2017. Interestingly, this period coincides with the outbreak of ceftriaxone resistant S. typhi in Hyderabad during 2016-2017 [12,13]. However, there has been an overall decrease in resistance rates of first line antibiotics from 2012 to 2018. Qamar et al. reported a similar trend of reduction in first-line antibiotic resistance among cases of S. typhi from 2012 to 2014 in Hyderabad and Karachi [13]. There are reports of re-emergence of first-line antibiotic sensitivity in India and Nepal as well [5,8]. Explanation for this phenomenon is long-term reduced use of first-line antibiotics resulting in re-emergence of their sensitivity [13]. Moreover, there has been a fall in ciprofloxacin resistance rate and a slight rise in cephalosporin resistance rate among S. typhi isolates from 2012 to 2018 in our study; a trend also reported by Qamar et al. [13]. Over the last decade, ciprofloxacin use for enteric fever has been decreasing whereas cephalosporins use has been proportionately increasing thus explaining this trend.

The findings of this study recommend the use of third-generation cephalosporins as empirical agents of choice and for treatment of MDR cases of enteric fever; cefixime for oral treatment and ceftriaxone for parenteral therapy. Imipenem use should be reserved for the treatment of XDR cases of enteric fever. Azithromycin and ciprofloxacin should not be routinely used for empiric treatment owing to high drug resistance rates. Decreasing trend in first-line antibiotic resistance is promising; it may lead to re-emergence of their sensitivity in the coming years. Therefore, antibiogram surveillance should be routinely performed to monitor antibiotic resistance patterns and to guide empirical treatment. Antimicrobial stewardship programs should be implemented to combat the growing problem of antibiotic resistance. General public should be educated about the risk factors and routes of transmission for enteric fever and encouraged to adopt measures of primary prevention. Health authorities should consider Salmonella vaccination campaigns to lessen the disease burden.

## Conclusions

S. typhi accounted for most of the cases of enteric fever in the present study. Male predominance and seasonal variation with highest incidence during months of summer and monsoon were observed. Most of the S. typhi isolates were resistant to traditional first-line antibiotics and ciprofloxacin. Antibiotic resistance rates were lower in S. paratyphi isolates. This study recommends third-generation cephalosporins for empirical treatment and for treatment of MDR cases of enteric fever. Imipenem should be reserved for treatment of XDR Salmonella cases. Declining first-line antibiotic resistance rates is promising and it may lead to re-emergence of first-line antibiotic sensitivity at some point in future. Therefore, routine surveillance of Salmonella antibiogram and antibiotic stewardship is the need of the hour. Public health education campaigns and salmonella vaccination programs should be considered for primary prevention of the disease.
